# Clustered metallothionein genes are co-regulated in rice and ectopic expression of *OsMT1e-P* confers multiple abiotic stress tolerance in tobacco via ROS scavenging

**DOI:** 10.1186/1471-2229-12-107

**Published:** 2012-07-10

**Authors:** Gautam Kumar, Vaishali Panjabi-Sabharwal, Sumita Kumari, Rohit Joshi, Ratna Karan, Shweta Mittal, Sneh L Singla Pareek, Ashwani Pareek

**Affiliations:** 1 Stress Physiology and Molecular Biology Laboratory, School of Life Sciences, Jawaharlal Nehru University, New Delhi, 110067, India; 2 Plant Molecular Biology, International Centre for Genetic Engineering and Biotechnology, New Delhi, 110067, India

## Abstract

**Background:**

Metallothioneins (MT) are low molecular weight, cysteine rich metal binding proteins, found across genera and species, but their function(s) in abiotic stress tolerance are not well documented.

**Results:**

We have characterized a rice MT gene, *OsMT1e-P*, isolated from a subtractive library generated from a stressed salinity tolerant rice genotype, Pokkali. Bioinformatics analysis of the rice genome sequence revealed that this gene belongs to a multigenic family, which consists of 13 genes with 15 protein products. *OsMT1e-P* is located on chromosome XI, away from the majority of other type I genes that are clustered on chromosome XII. Various members of this MT gene cluster showed a tight co-regulation pattern under several abiotic stresses. Sequence analysis revealed the presence of conserved cysteine residues in OsMT1e-P protein. Salinity stress was found to regulate the transcript abundance of *OsMT1e-P* in a developmental and organ specific manner. Using transgenic approach, we found a positive correlation between ectopic expression of OsMT1e-P and stress tolerance. Our experiments further suggest ROS scavenging to be the possible mechanism for multiple stress tolerance conferred by OsMT1e-P.

**Conclusion:**

We present an overview of MTs, describing their gene structure, genome localization and expression patterns under salinity and development in rice. We have found that ectopic expression of OsMT1e-P enhances tolerance towards multiple abiotic stresses in transgenic tobacco and the resultant plants could survive and set viable seeds under saline conditions. Taken together, the experiments presented here have indicated that ectopic expression of OsMT1e-P protects against oxidative stress primarily through efficient scavenging of reactive oxygen species.

## Background

Metallothioneins (MTs) are low molecular weight, cysteine-rich metal chelators with an ability to bind heavy metal ions. MTs are able to bind a variety of metal ions by the formation of mercaptide bonds between numerous Cys residues (present in the proteins) and the metal**,** and thus contribute to metal detoxification by buffering cytosolic metal concentration [1]. MTs typically contain two metal-binding, cysteine-rich domains that give these metalloproteins a dumbbell conformation. They are widely distributed in animals, plants, fungi as well as cyanobacteria. Based on sequence similarities and their phylogenetic relationships, MTs have been broadly classified into three types [2-4]. Class I MTs are widespread in vertebrates and have 20 conserved cysteine residues giving them the dumbbell conformation. Class II MTs do not have this strict arrangement of cysteine residues and are widespread in plants, fungi and invertebrates. On the other hand, phytochelatins, which are enzymatically synthesized metal binding peptides, are described as Class III MTs.

Plant MTs identified so far contain two cysteine-rich domains and a large spacer region (30–50 a.a. residues, devoid of cysteine) [5,6]. These MTs have only a few histidines, while their Cys content varies between 10 and 17 residues. On the other hand, the number of aromatic amino acids in plant MTs varies from none to several. Based on the distribution of cysteine residues, number of aromatic amino acids as well as length of the spacer region, plant MTs are further classified into four types, type 1 through 4 [1,6,7]. Analysis of various EST database shows that MTs are amongst the highly abundant transcripts in plants [8]. Recent studies have established important roles for plant MTs in fruit development, root development and suberization besides heavy metal tolerance [9-12]. Furthermore, the role of plant MTs in abiotic stresses such as oxidative, dehydration, senescence as well as hormonal alterations have also been shown [10,13,14]. The antioxidant function of MTs is attributed to the presence of a large number of cysteine residues, which besides metal binding are also capable of ROS scavenging [6]. Recently, a type 1 MT from mustard i.e. LSC54 has been reported to be induced by ROS production [15]. Further, LSC54 has also been documented to be related to ROS imbalance during leaf senescence. Similarly, in rice, several type 1 and type 2 MTs have been found to play a direct role in antioxidation [16].

Abiotic stresses, a major factor in reducing plant productivity, are proving to be an increasing threat to agriculture. Development of genetically modified abiotic stress tolerant varieties may provide a solution to this problem [17]. Recently, we have characterized the molecular response of rice seedlings towards salinity stress based on their subtractive transcriptome profiling [18]. One of the key members of this response, *OsMT1e-P* – a type 1 MT isolated from a salinity tolerant genotype i.e. *O. sativa* cv. Pokkali has been reported to be induced strongly in response to salinity stress. In the present communication, we provide detailed investigations on *OsMT1e-P.* In essence, we have found that *OsMT1e-P* is transcriptionally induced in response to salinity stress. Ectopic expression of OsMT1e-P in transgenic tobacco (under the control of 35 S constitutive promoter) provides stress tolerance against salinity, drought, cold, heat and heavy metals (Cu^2+^ and Zn^2+^). OsMT1e-P over expressing plants accumulate lower amount of ROS (H_2_O_2_) under salinity stress. Further, we also show that at least five members of type 1 MT are tightly clustered on the distal arm of chromosome XII which show co-regulation in response to various abiotic stress conditions. Based on these results, we propose that *OsMT1e-P* may serve as an important “candidate gene” for raising ‘multi-stress tolerant’ crops.

## Results and discussion

### MT gene family in rice: highly conserved gene family with 5 members clustered on chromosome XII

The whole genome analysis of rice (*O. sativa* TIGR database ver. 6.1) revealed the presence of 13 MT genes and 15 protein products in *O. sativa* sp. Japonica which have been further subdivided into four types (Table 1). In *Arabidopsis,* nine MT members have been reported previously [4]. The presence of multiple metallothionein genes may indicate their diverse role in plant responses. Their high numbers in plants may also be related to their distinct and overlapping biological roles by the regulation of gene expression and signaling [14]. The classification of these MT proteins was performed based on the conserved cysteine residue positions [6]. The members of the MT protein family were named as OsMT, where the first two initials represent the name of the organism viz. ‘Os’ for *Oryza sativa* followed by metallothionein abbreviated as ‘MT’. The numeral (1–4) represents the type of MT and alphabet represents the member number identified, numerals following the alphabet represent alternatively spliced products. There are eight members in type 1-OsMT1 *(*a1, a2, b, c, d, e, f and g); type 2 has five members (OsMT2a, 2b1, 2b2, 2c and 2d); whereas type 3 and type 4 have only one member each (Table 1). 

**Table 1:** Members of metallothionein family in rice

**Earlier proposed name**	**Gene**	**Protein name**	**Tigr ID**	**Locus**	**Coordinate**	**Amino acids**
OsMT-I-4a	OsMT1a	OsMT1a1	12012.m07613	LOC_Os12g38270.1	23467876 – 23468536 (+)	108
		OsMT1a2	12012.m56497	LOC_Os12g38270.2	23467876 – 23468352 (+)	82
OsMT-I-1b	OsMT1b	OsMT1b	12003.m07212	LOC_Os03g17870.1	9936480 – 9937064 (+)	73
OsMT-I-4b	OsMT1c	OsMT1c	12012.m73910	LOC_Os12g38051.1	23350565 – 23349930 (-)	80
OsMT-I-4c	OsMT1d	OsMT1d	12012.m07616	LOC_Os12g38300.1	23481607 – 23482236 (+)	79
OsMT-I-1a	OsMT1e	OsMT1e	12011.m80109	LOC_Os11g47809.1	28269831 - 28270196 (+)	75
	OsMT1f	OsMT1f	12012.m07587	LOC_Os12g38010.1	23319560 – 23318879 (-)	79
	OsMT1g	OsMT1g	12012.m07615	LOC_Os12g38290.1	23479046 – 23479680 (+)	77
	OsMT2a	OsMT2a	12001.m07196	LOC_Os01g05650.1	2691774 – 2690331 (-)	83
OsMT-I-2c	OsMT2b	OsMT2b1	12005.m28027	LOC_Os05g02070.3	584010 – 584488 (+)	85
		OsMT2b2	12005.m27672	LOC_Os05g02070.4	584010 – 584488 (+)	58
OsMT-I-2b	OsMT2c	OsMT2c	12001.m13456	LOC_Os01g74300.1	43374103 – 43374519 (+)	81
	OsMT2d	OsMT2d	13101.m00538	LOC_Os01g05585.1	2665458-2664412 (+)	65
OsMT-I-3a	OsMT3a	OsMT3a	12001.m07661	LOC_Os01g10400.2	5475423 – 5476251 (+)	61
OsMT-II-1a	OsMT4	OsMT4	12010.m06732	LOC_Os10g39610.2	20838860 – 20839209 (+)	88

The multiple sequence alignment of the various rice MT protein sequences (based on *in silico* predictions) with the protein sequence of MT gene cloned in our laboratory from Indica rice genotype Pokkali, OsMT1e-P [Genbank: EU684548] has been shown in Additional file 1: Figure S1A*.* Based on this analysis, it could be concluded that rice MTs share a very high degree of conservation (as high as 98% within their respective types). Two of them i.e. OsMT1a1 and OsMT1a2 have an additional stretch of 17 amino acids which is not present in any other type 1 members. The rooted phylogenetic tree of MTs reported from *Arabidopsis* and rice (based on the amino acid sequence) showed a very close relationship between them (Additional file 1: Figure S1B). OsMT1e-P of Pokkali was observed to have 100% identity with OsMT1e member of *O. sativa* sp. Japonica and both of them clustered with other type I members.

**Figure 1:**
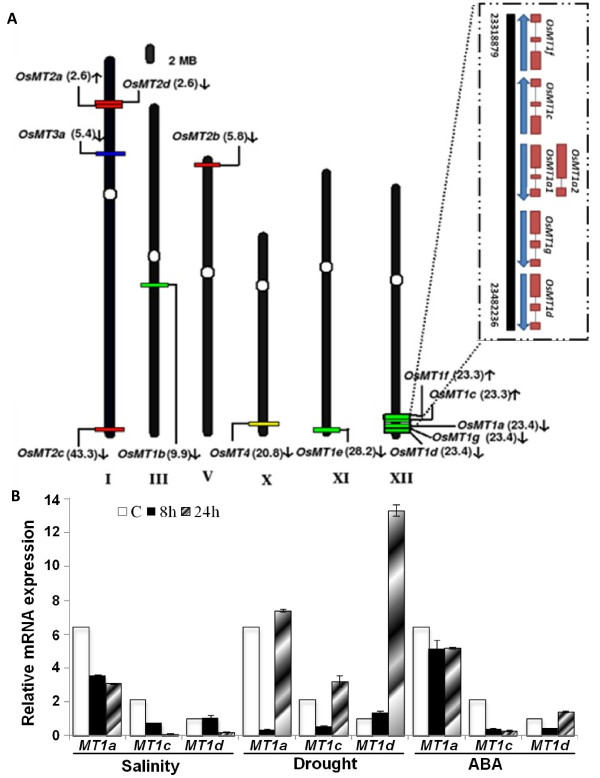
**Graphical (scaled) representation of location of putative genes for* OsMTs *on* O. sativa *chromosomes and qRT PCR analysis of clustered MT genes localized on chromosome XII.** (**A**) The centromeres on chromosomes have been marked in circles. The position of first exon of genes (in Mb) has been marked in the parentheses along with their names at same location on chromosomes. Arrow marks the direction of the ORF specific to the * OsMT * genes. Enlarged view of a portion of chromosome XII (inset) shows a cluster of MT genes and their distribution revealing the striking similarity in the gene structure of the members. Intron/exon(s) are shown as thin and thick line, respectively. (**B**) Real-time PCR analysis of * OsMT1a, OsMT1c, * and * OsMT1d * expression in shoots of 4-day old rice seedlings under various abiotic stresses such as salinity (200 mM NaCl), drought (air drying) and ABA (100µM) for various durations i.e. 8 h or 24 h. Expression of various * MTs * have been shown relative to that of the * MT1d * which is the lowest expressed gene under control conditions. The controls were grown in half strength Yoshida medium. Data are means ± SE. Each data set represents an average of minimum three separate experiments.

Chromosomal localization of the various MT members in *O. sativa* is shown in Figure 1A. These *OsMT*s were found to be scattered on only six chromosomes i.e. I, III, V, X, XI and XII. Three members of the type 2 MT proteins - *OsMT2a* (in antisense orientation), *OsMT2d* and *OsMT2c* were located on chromosome I, while the fourth, *OsMT2b*, was located on chromosome V. The type 3 and type 4 classes, having a single member each, were located on chromosome I and chromosome X, respectively. Thus, as far as chromosomal localization is concerned, no specific arrangement of MTs belonging to type 2, 3 and 4 were noticed. In contrast, most of the type 1 MT genes were found to be tightly clustered together (within a segment of 150 kb) on chromosome XII (from 23.3 - 23.4 Mb region; Figure 1A). Two of these genes namely *OsMT1f* and *OsMT1c* were found to be on the antisense strand, while other three *OsMT1a*, *OsMT1g* and *OsMT1d* were found to be on the sense strand. *In-silico* analysis of various *OsMT1* genes revealed that they share highly identical gene structure i.e. three exons and two introns per gene, except the alternatively spliced *OsMT1a2* which shows the presence of two exons and a single intron (Figure 1A inset). This unique arrangement of MTs in near vicinity on chromosome XII and their high sequence conservation possibly indicate that the present situation is the result of segmental duplication of chromosome. To test this hypothesis, we performed sequence analysis of these regions of chromosomes using Mummer software. This analysis indicated that certain regions of chromosome XII (having MT genes) may have got duplicated and diverged further during the course of evolution. Further, analysis of various orthologous members with respect to the *OsMT1e* gene showed that *OsMT1a, OsMT1b, OsMT1c, OsMT1f* and *OsMT1g* belong to the same orthologous group. Thus, it can be inferred that *OsMT1e* gene might have got duplicated on chromosome XII at several instances and thus got further diverged from each other. Dot plot analysis (Additional file 2: Figure S2) of the region showed that these genes have diverged enough but during this process, they have preserved their metallothionein signatures as well. Earlier, analysis of genomes of maize, rice and wheat has led to the identification and characterization of shared duplications between rice and wheat [19,20]. Interestingly, MT genes in rice were not observed to be in synteny with maize, sorghum, wheat and barley. However, the extensive duplication and evolution of type 1 MTs in rice reinforces their importance in plants. 

### Quantitative real-time PCR analysis indicated co-regulation of clustered metallothionein genes

Since *OsMT1a*, *OsMT1c*, *OsMT1d*, *OsMT1f* and *OsMT1g* genes were found tightly clustered on chromosome XII, we wanted to check if these genes are ‘transcriptionally co-regulated’ as well. For this purpose, qRT-PCR was carried out with cDNA prepared from shoots of rice seedlings subjected to salinity, drought and ABA stress. Figure 1B shows transcript abundance for three of these genes namely *OsMT1a*, *OsMT1c* and *OsMT1d*. This analysis indicated that these three “tightly placed” genes are down regulated under salinity stress as well as exogenous ABA application while up regulated after 24 h of drought stress (barring *OsMT1a* and *OsMT1c* where down regulation in transcript was seen just after 8 h of stress). However, we could not quantify the transcripts for other two genes namely *OsMT1g* and *OsMT1f* which may be due to the extremely low abundance of transcripts of these genes under the tested conditions (the low abundance of these transcripts was also confirmed through the analysis of rice MPSS database). This data clearly indicates that these clustered metallothionein genes are co-regulated under various stresses in the shoots of rice seedlings. Similar observation has been made previously where co-expression of nuclear genes, coding for different subunits of photosystem or proteins involved in the transcription/translation of plastome having overlapping functions, has been documented as a novel mechanism for co-ordinated regulation of gene expression [21].

### Salinity and development regulated expression of OsMT1e-P

**Figure 2:**
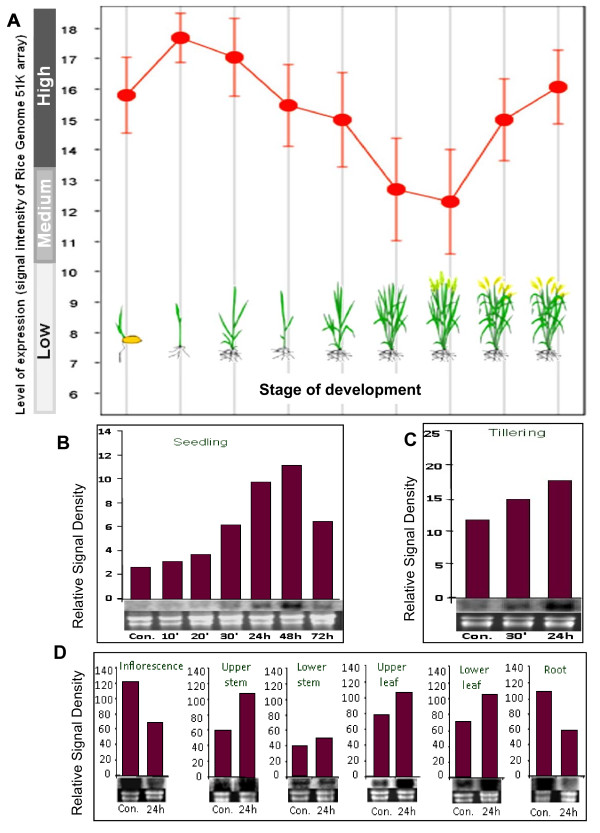
**Salinity and development regulated expression of* OsMT1e-P *in rice (Pokkali) grown in green house under standard agronomic practices.** (**A**) Expression pattern of * OsMT1e-P * at various developmental stages of * Oryza sativa * based on microarray data (www.genevestigator.com). The expression data shown here is normalized, high quality and manually curated data obtained from several hundreds of independent experiments. The stage of development of plant is also shown diagrammatically in the figure. (**B**) Time course RNA gel blot analysis of * OsMT1e-P * transcripts in shoots of Pokkali rice seedlings stressed with 200 mM NaCl for 10′, 20′, 30′, 24 h, 48 h or 72 h. (**C**) Time course RNA gel blot analysis of * OsMT1e-P * transcripts in shoots of Pokkali rice tillers stressed with 200 mM NaCl for 30′ or 24 h. (**D**) RNA gel blot analysis of the * OsMT1e-P * expression profile in various tissues of mature rice plants (stressed with 200 mM NaCl for 24 h), grown in green house. The 0 h time point served as the control in all the analyses. The RNA gel blot analysis was performed twice with similar results. The EtBr stained RNA gel is shown below each RNA blot as loading control. The scanned intensity of each band is shown above the corresponding blot in the form of a histogram.

Plant stress responses often mimic certain normal developmental processes [22]. Interaction between development and environmental conditions implies that some genes must be co-regulated by both environmental and developmental cues. Since *OsMT1e-P* was found to be induced strongly in response to salinity stress [18], detailed investigations on its transcript regulation and functional validation was carried out. To see the effect of development on transcript accumulation of *OsMT1eP* in rice plants, we looked into publically available microarray data. Very high transcript abundance for *OsMT1eP* was noted at two most stress sensitive stages of plant development i.e. seedling and reproductive stage (Figure 2A). The vegetative and tillering stages showed relatively low abundance for this transcript. These results are in line with the existing literature where various stress responsive genes have been shown to have developmental regulation and a stage specific pattern of their induction has been reported [22,23]. 

It has been established in literature that both ‘very early’ and ‘late’ responses associated with salinity stress have their own significance. The very early response is generally the ‘shock response’ while the late responses may often have genetic basis [18,24]. Since the *OsMT1e-P* gene was obtained in a salinity stress specific subtractive library [18], RNA gel blot analysis was carried out to study the effect of salt stress on transcript abundance in rice seedlings. We have carried out this transcript abundance analysis twice but data from only one such representative set is presented in Figure 2B. Transcripts for *OsMT1eP* could be detected in rice seedlings even under non-stress conditions which were further induced during salinity stress (10’, 20’ or 30’). The transcript levels further increased in response to 24 h of stress treatment and continued to increase up to 48 h of stress (as high as 6 folds - as compared to non stress conditions, Figure 2B). Even after 72 h of stress, induced levels of *OsMT1e*-P could be detected, though lesser as compared to that in response to 48 h of stress.

The salinity stress inducibility of *OsMT1e-P* was also observed at the tillering stage as its transcript increases within 30’ and 24 h of salinity stress (Figure 2C). In this case, we could again see a clear time-dependent transcript accumulation pattern in response to salinity stress. We have further extended this analysis to various tissues of mature rice plants grown under standard agronomic practices. In Figure 2D, it could be seen that the various tissues of the mature rice plants maintain differential levels for *OsMT1e*-*P* transcripts which in turn are differentially regulated by salinity stress. All the tissues analyzed in this study i.e. inflorescence, upper stem, lower stem, upper leaf, lower leaf and roots showed a constitutive level of expression for *OsMT1e*-*P*, with roots and inflorescence showing the highest levels. Moreover, the constitutive levels of transcripts were higher at maturity stage than seedling and tillering stage. *OsMT1e*-*P* was further found to be induced by salinity in all the tissues except roots and inflorescence, where down regulation of the transcripts could be seen in response to salinity stress. It is interesting to note that both the apical tissue of the mature plant behave differently from all other tissue.

The expression patterns obtained in mature plant tissues are in agreement with the previous studies where type 1 MT-like transcripts have been detected primarily in roots, senescing leaves, stems, leaves and flowers [25-27]. Several rice MT1 and MT2 genes show high transcript accumulation in roots, seedlings as well as stem [26-28]. Probably, the down-regulation of *OsMT1e-P* expression in the early phase of stress results in an oxidative burst phase, which potentiates ROS to function as a signal in salinity stress tolerance. Roots are the most appropriate site for initiating stresses response as they are the primary site of perception and injury for several types of water-limiting stresses, including salinity and drought. Under many circumstances, it is the stress-sensitivity of the root that limits the productivity of the entire plant. A high level of *OsMT1e-P* induction was detected in the leaves, while relatively low induction was detected in stem (Figure 2D). Leaves are more sensitive than stem due to the presence of highly developed photosynthetic apparatus which is an active site for ROS production [29] and hence metallothionein induction in leaves is important for protection under abiotic stress.

### OsMT1e-P transgenic plants show enhanced tolerance to multiple abiotic stresses including salinity

The response of plants to abiotic stress consists of a coordinated function of several biochemical pathways to regulate cellular ion homeostasis, enabling the plant to show tolerance and yield stability under saline environments. Reports have suggested that although abiotic stress is a multigenic trait, stress tolerant plants could be produced by transgenic approaches by the transfer of a single or multiple genes. Several genes such as barley *HVA1*[30], rice *CDPK*[31], alfalfa *Alfin1*[32], tobacco *NPK1*[33], and *Brassica GlyI* and rice *GlyII*[34-37] have been expressed in transgenic plants to enhance their stress tolerance [38].

**Figure 3:**
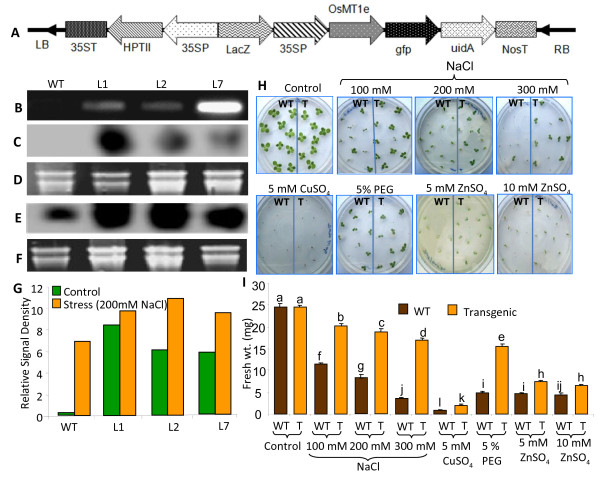
**Confirmation of* OsMT1e-P *transgenic tobacco plants and assessment of their tolerance towards salinity stress.** (**A**) Schematic representation of construct (pCAMBIA* OsMT1e-P *) used to ectopically express * OsMT1e-P * in tobacco plants. (**B**) PCR analysis of the wild type (WT) and T_0_ transgenic lines (L1, L2 and L7) to confirm the integration of the * OsMT1e-P * gene. (**C**) Northern blot analysis of * OsMT1e-P * transcript in shoots of seven days old WT and T_0_ transgenic lines (L1, L2 and L7) under unstressed condition. (**D**) Ethidium bromide stained RNA gel used as loading control for northern blot analysis of samples used in C. (**E**) Northern blot analysis of * OsMT1e-P * transcript in shoots of seven days old WT and T_0_ transgenic lines (L1, L2 and L7) under salinity (200 mM NaCl, 48 h) stress condition. (**F**) Ethidium bromide stained RNA gel used as loading control for northern blot analysis of samples used in E. (**G**) Histogram depicting signal intensity of * OsMT1e-P * transcript in WT and T_0_ transgenic lines (L1, L2 and L7) under unstressed and salinity (200 mM NaCl) stress conditions as obtained from C and E above. (**H**) Germination assay of *OsMT1e-P* transgenic seeds (T_1_) on MS media supplemented with various stressors. For this purpose, medium was supplemented with either NaCl (100 or 200 or 300 mM) or CuSO_4_ (5 mM) or PEG (5%) or ZnSO_4_ (5 mM or 10 mM). Seeds sown on plain MS medium served as control. (**I**) Histograms depicting fresh weight of WT and * OsMT1e-P * transgenic seedlings grown under various stress conditions. The seedlings were maintained under culture room conditions (28 ± 1^0^ C, 16 h light/8 h dark) and their growth was monitored for 15 days under different stresses and fresh weight of surviving seedlings was measured. The data represent means ± SE of three biological replicates (n = 3). Bars with different letters are statistically significant and those with the same letters are not significantly different (p < 0.05).

To establish the role of *OsMT1e-P* gene in abiotic stress tolerance, the full length gene cloned in pCAMBIA1304 vector (513 bp) was introduced into tobacco plants by *Agrobacterium* mediated transformation (Figure 3A). The preliminary screening of putative tobacco transformants was performed with tissue PCR, employing primers designed based on the T-DNA sequence that flanks the cloned *OsMT1e-P gene*. Three independently transformed T_0_ transgenic plants (L1, L2 and L7) were randomly selected for subsequent analysis. The integration of the *OsMT1e-P gene* into the tobacco genome was further confirmed by PCR analysis. A single band of 513 bp, corresponding to the *OsMT1e-P* cDNA, was observed in transgenic plants while no band was detected in the WT plants (Figure 3B). Northern blot analysis of wild type and T_0_ lines showed the presence of higher transcript levels in all the transgenic plants in comparison to WT under normal growth condition (Figure 3C, D). Moreover, increase in the transcript was observed in both WT and transgenic lines when subjected to salinity (200 mM NaCl, Figure 3E, F). These results show that a transcript signal corresponding to the size of *OsMT1e-P* transcript was detected in WT tobacco plants also, under stress conditions, when rice *OsMT1e-P* gene was used as probe. This indicated that endogenous gene for MT1e in tobacco gets up regulated under salt stress condition. Since it gets detected when rice *OsMT1e-P* was used as probe, it indicates that there is a good sequence homology between the two orthologs. Densitometry analysis showed that *OsMT1e-P* transcript abundance was higher in transgenic lines as compared to wild type maintained under control (no stress) as well as salinity stress conditions (Figure 3G).

Since, OsMT1e-P is also a putative metal binding protein, multiple stress tolerance ability of OsMT1e-P ectopic expressing lines (T_1_) were assayed at the seedling stage by transferring them to various concentrations of NaCl (salinity), PEG (dehydration), ZnSO_4_ and CuSO_4_ (heavy metal stress) and monitoring their growth for 15 days. All *OsMT1e-P* transgenic seedlings showed comparable growth in the absence of NaCl (Figure 3H). However, in the presence of mild salinity (100 mM NaCl), a strong difference in seedling growth was observed between WT and transgenic lines. When exposed to 200 mM NaCl, strong contrast was observed in growth pattern where the transgenic seedlings grew much better as compared to WT seedlings. WT seedlings just could not grow in the presence of higher levels of salinity (300 mM NaCl), but most of the seedlings of transgenic lines were able to grow well under similar conditions. Similarly, transgenic seedlings outperformed WT plants under 5% PEG or ZnSO_4_. However, in the presence of 5 mM CuSO_4_, the WT and transgenic lines behaved almost the same way where growth of the seedlings was almost arrested. Histograms depicting the differences in the fresh weight of transgenic *vis-a-vis* wild type tobacco lines show a clear advantage of OsMT1e-P ectopic expression, particularly under salinity, PEG and zinc stress (Figure 3I). Stress mitigating capacity of several plant metallothionein genes is mediated through their metal binding capacity [11,13,14]. When released from metallothionein proteins, Zn^2+^/Cu^2+^ ions become available for antioxidant metal binding enzymes e.g. Cu, Zn-superoxide dismutase and may also help in spatial regulation of oxidoreductive environment in the cell [39]. MTs have been reported to initiate Zn^2+^ mediated antioxidant response in mammals and fungi [40]. Improved growth and survival of *OsMT1e-P* transgenic plants under multiple stresses is proposed to be mediated through maintenance of redox balance and thereby reducing ROS induced injury.

Leaf disc assay has been shown to be one of the quick assays for assessing the tolerance of plants towards salinity stress [35]. We also performed this assay with T_1_ plants in the presence of high salinity (200 mM and 300 mM NaCl). All the transgenic lines (L1, L2 and L7) showed considerably better chlorophyll retention under high salinity (after five days of stress), while WT lines showed excessive bleaching under similar conditions (Additional file 3: Figure S3A). To explore if the ectopic expression of *OsMT1eP* is contributing towards better ion homeostasis, ionic measurements were carried out in leaves of the salt-stressed WT and transgenic plants. This study revealed that there was relatively less disturbance in the ion balance in transgenic plants as compared to the WT plants under salinity stress. Under control conditions, a K^+^/Na^+^ ratio in the range of 11.5 to 12 was observed for both WT and transgenic plants while a drastic decline was observed in WT when subjected to 200 mM NaCl (4.36) and 300 mM NaCl (0.74) for 5 days (Additional file 3: Figure S3B, statistically significant differences have been marked as different alphabets on the top of the bars). In contrast, the transgenic lines maintained this ratio between 7.6-9.9 and 3.3-4.5 under 200 mM and 300 mM NaCl, respectively. Maintenance of K^+^/Na^+^ homeostasis is considered as an important aspect of salinity tolerance in plants and the higher K^+^/Na^+^ levels are very well correlated with enhanced salinity tolerance [35,41,42].

### Reduced accumulation of H_2_O_2_ in the transgenic lines shows their enhanced antioxidant activity

**Figure 4:**
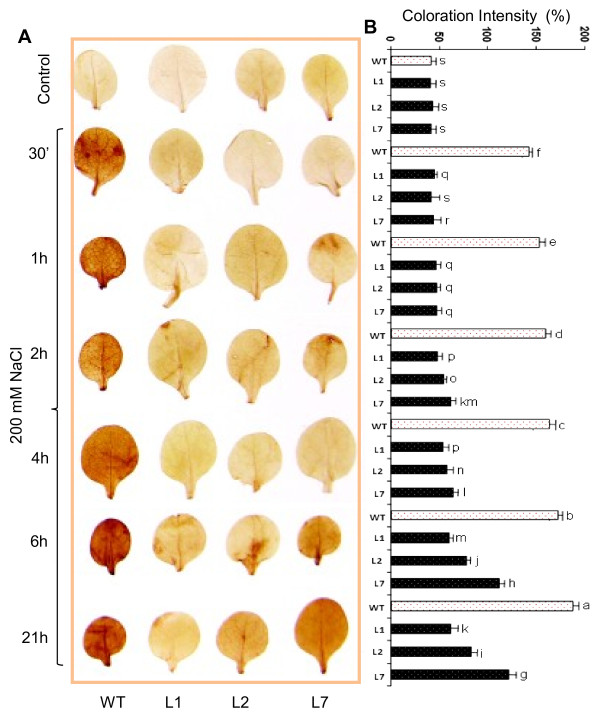
**Assessment of relative H**_**2**_**O**_**2 **_** levels in tobacco plants under salinity stress.** (**A**) Constitutive and salinity stress (200 mM NaCl) induced levels of H_2_O_2_ in leaves of WT as well as *OsMT1e-P* ectopically expressing lines (T_1_ - 15-d-old plants) were visualized by DAB staining. (**B**) Histograms depicting relative quantity of H_2_O_2_ in these leaves in terms of their colouration intensity. The data represents the means ± SE of three biological replicates (n = 3). Bars with different letters are statistically significant and those with same letter are not significantly different (p < 0.05).

Abiotic stresses like drought, high salinity and low temperature ultimately lead to oxidative stress due to the disturbance in ROS production and scavenging, leading to generation of reactive oxygen species (ROS), such as H_2_O_2_[43-45]. In biological systems, ROS are highly reactive and toxic. They partially reduce or activate derivatives of oxygen and thus damage DNA, proteins and carbohydrates, resulting in cell death, ultimately [46,47]. ROS also cause lipid peroxidation and cell membrane damage and their levels can reflect the degree of damage to cellular components [48]. Because ectopic expression of OsMT1e-P improved the salt stress tolerance of transgenic tobacco plants, we examined whether OsMT1e-P functions in stress tolerance through detoxification of ROS. For this purpose, leaves of the WT and transgenic lines (T_2_), subjected to salt stress for 30 min, 1 h, 2 h, 4 h, 6 h and 21 h were stained with DAB to reveal *in planta* accumulation of H_2_O_2_. Histochemical staining showed that under control conditions, transgenic lines had ROS levels similar to WT. Salinity stress resulted in notable increase in ROS levels in WT within 30 min as these leaves showed intense brown colored polymer accumulation in midribs and veins (Figure 4A). However under similar condition, the leaves of transgenic lines showed remarkably less accumulation of brown colored polymer implying less ROS production. This contrast in H_2_O_2_ levels between WT and transgenic lines was noticed even after longer durations of salinity stress (4-6 h). The visual observation of the quantitative difference in the H_2_O_2_ accumulation was reconfirmed by densitometric scanning of the DAB stained leaves (Figure 4B). After 21 h of stress, transgenic lines also show a slight increase in H_2_O_2_ level but still much lower than that of WT plants (the differences were statistically significant as shown by different alphabets on the bars in the Figure 4B). Out of the three transgenic lines studied here, line L1 was found to be relatively more tolerant to salinity stress than the other two. As ROS levels during stress greatly relies on the homeostasis between ROS generation and removal [49], accumulation of less ROS in the transgenic lines seems to indicate that ROS scavenging systems in these plants might work more effectively as compared to WT. MTs can protect plants by participating in signaling (early response) or adaptation (late response). Up regulation of the antioxidant thiol protein, MT during late phases of oxidative stress, helps in mitigating stress by scavenging the ROS probably through the oxidation of cysteine thiols [50]. Recently, a number of reports have demonstrated MTs as efficient ROS scavengers in animals [51-53]. Based on this analysis, we propose that OsMT1e-P may enhance plant stress tolerance through the up regulation of antioxidative enzymes and the reduction of ROS. 

### Ectopic expression of OsMT1e-P in tobacco provides multiple stress tolerance in subsequent generations

**Figure 5:**
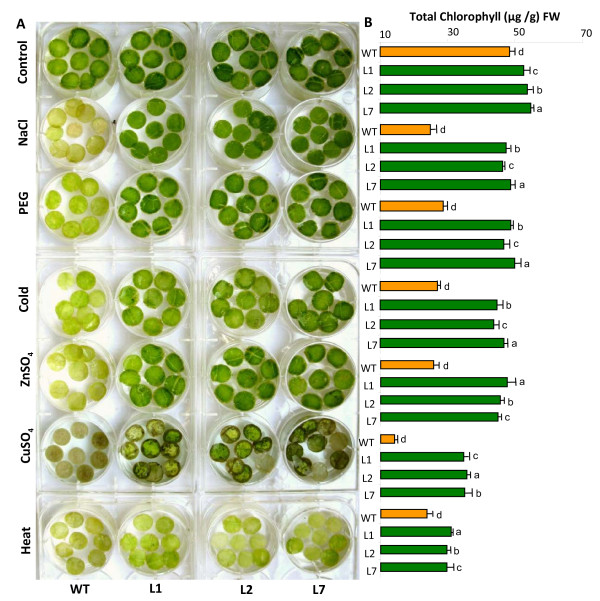
**Assessment of* OsMT1e-P *transgenic tobacco plants for their tolerance towards various abiotic stresses and heavy metal toxicity.** (**A**) Floating leaf disc senescence assay for abiotic stress tolerance in transgenic tobacco plants (T_2_). Experiments were performed on three transgenic lines (L1, L2 and L7) subjected to either 42^0^ C for heat stress, 10 mM CuSO_4,_ 10 mM ZnSO_4,_ 4^0^ C for cold stress, 5% PEG or 200 mM NaCl stress in Hoagland medium. Heat stress was given for 8 h only while other stresses were given for 5 days. Leaf discs floated in Hoagland medium served as the experimental controls. (**B**) Histogram depicting chlorophyll content retained in corresponding leaf discs shown in A. The data represent mean ± SE of three biological replicates (n = 3). Bars with different letters are statistically significant and those with same letter are not significantly different (p < 0.05).

Since we found that transgenic tobacco seedlings (T_1_) outperform the WT plants in the leaf disc assays under salinity stress (Additional file 3: Figure S3), we decided to extend the analysis to T_2_ generation and also various abiotic stresses such as dehydrations (PEG), cold, heavy metals (ZnSO_4_ and CuSO_4_) and high temperature. Leaf disc assays showed a clear advantage of transgenic lines over WT plants in overcoming the deleterious effects of NaCl toxicity. The WT plants showed rapid senescence and extensive bleaching reflecting symptoms of injury due to stress, while the transgenic lines performed better under similar conditions (Figure 5A). Chlorosis was also less in transgenic lines under PEG stress (dehydration stress), cold stress, ZnSO_4_, and copper (CuSO_4_) while the WT was completely bleached under similar conditions. However, under high temperature stress, even the transgenic lines showed injury symptoms (chlorophyll depletion) but were still better than WT plants. The results of leaf disc assay were confirmed biochemically by estimating the chlorophyll content. There was nearly complete loss of chlorophyll in the WT plants, whereas the OsMT1e-P transformants showed relatively less reduction in total chlorophyll content at 200 mM NaCl (Figure 5B). Similar observations were made in response to other stresses and these results were also validated statistically as shown in different alphabets on top of the bars in Figure 5B. These observations establish a positive relationship (though not absolute) between the ectopic expression of OsMT1e-P and salinity tolerance, dehydration stress as well as heavy metal tolerance (Zn^2+^ and Cu^2+^) in leaf tissues. Another rice type I metallothionein, OsMT1a has been reported to provide tolerance against drought and oxidative stress besides being involved in Zn^2+^ homeostasis and its over expression leads to higher Zn^2+^ accumulation in rice seeds [13]. In *Arabidopsis*, all 4 types of MT proteins provide Cu^2+^ tolerance in yeast *cup1* mutant [12]. Similar metal toxicity alleviation has been reported in *Arabidopsis* where the *MT1* knockdown lines were hypersensitive to Cd^2+^ and accumulated several fold lower levels of Cd^2+^, and Zn^2+^ than WT [4]. 

### OsMT1e-P transgenic plants flower normally and set viable seeds under high salinity

**Figure 6:**
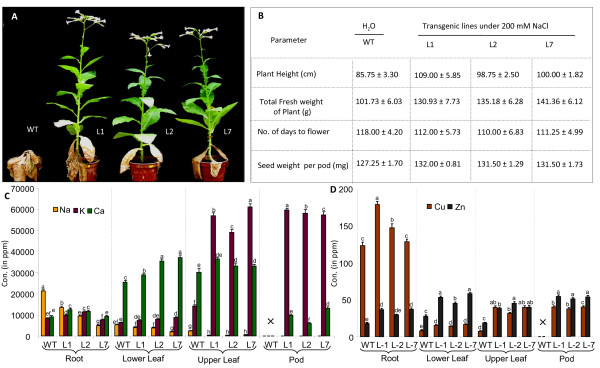
**Comparison of various yield parameters of WT and* OsMT1e-P *ectopically expressing tobacco plants, continuously grown under saline conditions.** (**A**) Growth of WT and * OsMT1e-P * tobacco plants grown in the presence 200 mM NaCl as assessed visually (**B**) Various growth and yield parameters of the WT and * OsMT1e-P * tobacco plants grown in the presence of water or 200 mM NaCl, respectively. (**C**) Na^+^, K^+^ and Ca^2+^ (**D**) Cu^2+^ and Zn^2+^ content [conc. as ppm], in various tissues of the * OsMT1e-P * ectopically expressing transgenic plants grown under continued presence of 200 mM NaCl, measured using EDXRF analysis. For each analysis, roots, old leaf, young leaf, or pods were collected from three different plants of each line. Similar data for WT plants could not be obtained as these plants did not grow further in the presence of 200 mM NaCl. The data represents mean ± SE of three biological replicates (n = 3). Bars with different letters are statistically significant and those with same letter are not significantly different (p < 0.05).

Since *OsMT1eP* seedlings were found to perform better than WT seedlings in response to salinity stress, we decided to test the performance of these plants under extended durations of stress. For this purpose, we used saline water for watering of plants till maturity. We found that while transgenic plants grew normally, flowered and produced seeds even in the presence of 200 mM NaCl, WT plants exhibited stunted growth, failed to flower and completely senesced before reaching maturity (Figure 6A). Several growth parameters viz. plant height, fresh weight, days to flower and pod weight of transgenic plants grown under salinity stress were compared with the similar parameters obtained from WT plants irrigated with water instead of salinity (WT plants could not sustain growth under saline conditions). Plant height and fresh weight measurements clearly suggests the capacity of *OsMT1e-P* transgenic plants in retaining higher biomass than WT under saline conditions or even WT plants under non saline conditions (Figure 6B). Flowering time was slightly delayed (2–7 days) in *OsMT1e-P* transgenic plants grown under stress *vis-à-vis* WT plants maintained under control conditions. However, it did not affect the seed setting and the overall yield of transgenic plants growing under salinity stress and the yield was comparable (or even higher) to that obtained from WT plants growing under non stress conditions (Figure 6B). Accumulation of various elements was analyzed in *OsMT1eP* transgenic plants using energy dispersive X-ray fluorescence (EDXRF) spectrometry which is a very useful technique for the identification of trace elements from the plant tissues [54]. OsMT1e-P ectopically expressing plants accumulate negligible Na^+^ as well as heavy metals Cu^2+^ and Zn^2+^ in upper leaf and pods as compared to older leaves and roots (Figure 6C, D). High accumulation of Na^+^ and Cu^2+^ in root indicates the ability of OsMT1e-P plants to use roots and lower leaves as sink thereby restricting the mobilization of deleterious amounts of these elements in the upper vegetative tissue and pods. These parameters thus clearly establish a positive correlation of *OsMT1eP* ectopic expression with salinity and heavy metal tolerance in tobacco plants. 

## Conclusions

Expression of *OsMT1e-P* gene is regulated by multiple abiotic stresses in a development and organ specific manner and is therefore an important candidate gene for abiotic stress tolerance. It is proposed that OsMT1e-P helps in detoxification and cellular repair while maintaining the cellular homeostasis via ROS scavenging directly or indirectly via other antioxidants. However, further studies are warranted to work out the precise mechanism to link their metal binding affinity with abiotic stress tolerance. OsMT1e-P probably interacts with other physiological processes in the cell for selective uptake and sequestration of ions during salinity stress.

## Methods

### Isolation of cDNA clone and sequencing

The *OsMT1e-P* clone (acc. no. EU684548) was isolated during subtractive hybridization of differentially expressed cDNAs from 4-day old seedlings of *O. sativa* L. cv Pokkali treated with 200 mM NaCl for 30 min [18].

### Sequence analysis of rice MT genes

The MT sequence protein profile was built using MT sequences obtained from NCBI database using psi-BLAST [55]. The sequences thus obtained were used to make profile using hmmbuild program of HMMER2 package (version 2.3.2; http://hmmer.janelia.org). This profile was further used for searching the presence of MT proteins in rice [TIGR version 6.1]. The sequences were aligned using MUSCLE [56] and the parsimonious tree was plotted using the alignment with Phylip package (version 3.6) with default parameters [57]. Pairwise alignment of the *OsMT* genomic sequences were carried out using Needle program of EMBOSS package (5.0.0) [58]. Mummer software package (v 3.20) [59] was used for analyzing the duplication of genes present on chromosome XII. The final figures for alignment were prepared using Jalview [60].

### Expression analysis based on microarray data

Genevestigator 4 (http://www.genevestigator.com) was used to fetch the expression data for *OsMT1eP* using default parameters [23]. The gene query was the LOC (Os.3445.1.S1_at). The output of the analysis was exported in the pdf format for presentation.

### Plant material and stress treatments for transcript analysis of OsMT1e-P under various conditions

For the analysis of transcript abundance under various stress conditions at the seedling stage, rice seeds were rinsed with distilled water and germinated in a hydroponic system for 7-days in half strength Yoshida medium. Salinity stress treatment was given by transferring the seedlings to 200 mM NaCl solution for 10 min, 20 min, 30 min, 8 h, 24 h, 48 h, and 72 h. Further, seedlings were exposed to drought/dehydration (air drying), and ABA (100 μM) for 8 h and 24 h. The untreated samples were taken as control.

Leaves of field grown plants at the tillering stage, while in the mature plant, tissues from various plant parts viz. stem and leaves (upper and lower), inflorescence and roots subjected to 200 mM salinity stress for 30 min or 24 h in ½ Yoshida medium and untreated samples were taken as control [18].

### Total RNA isolation, mRNA purification and cDNA synthesis

Total RNA was isolated using Raflex solution (Bangalore Genei) according to the manufacturer’s instructions. The concentration of RNA was determined using spectrophotometer and its integrity was checked on agarose gel. Enrichment of polyA^+^ RNA and cDNA synthesis was carried out as described earlier [61].

### Real-time PCR

Primers for real time PCR analysis of the *OsMT* gene members were designed using Primer Express 3.0 software (PE Applied Biosystems, USA). The sequences for these primers are given in additional file 4: Table S2 and real time PCR was performed as described earlier [61]. The specificity of amplification was tested by dissociation curve analysis and agarose gel electrophoresis. The expression of each gene in different RNA samples was normalized with the expression of internal control gene, *actin*. The mRNA levels for each candidate gene in different tissue samples were calculated relative to its expression in control seedlings using ΔΔCT method of SDS 1.4 software (Applied Biosystems). Three technical as well as biological replicates were analyzed for each sample (n = 3).

### Northern hybridization

Northern blot was prepared using 20 μg total RNA and probes were prepared by labeling the PCR amplicons with α^32^P-dATP using HexaLabel DNA labeling kit (Fermentas Life Sciences, USA). Hybridization, washing and scanning of RNA blots were carried out as described [18].

### Construction of plant transformation vector and generation of transgenic plants

For ectopic expression of *OsMT1e-P,* the complete ORF (513 bp) was PCR amplified by using primers *OsMT1eP*-F1 (5′-GAAGATCTTCATGTCTTGCAGCTGTGGATC-3′) and *OsMT1eP*-R1 (5′-GACTAGTCTTAACAGTTGCAAGGGTTGC-3′), and cloned at the *Bgl*II and *Spe*I sites of plant expression vector pCAMBIA1304. For tobacco transformation, the pCAMBIA*OsMT1e-P* construct was mobilized into *Agrobacterium tumefaciens* (GV3101) by liquid nitrogen freeze-thaw method. Tobacco leaf discs (*Nicotiana tabaccum* L. cv *Xanthi*) were transformed using the standard protocol [62] and the transformants were selected on hygromycin (25 mg/L).

### Genomic DNA PCR of transgenic plants

Putative transformants were screened by PCR analysis using tobacco genomic DNA (from WT and various transgenic lines) as template and vector specific primers (forward primer 5'-CAAGACCCTTCCTCTATATAAG-3' and reverse primer 5'-CAAGAATTGGGACAACTCCAG-3'). The pCAMBIA*OsMT1e-P* vector was used as template for positive control.

### Testing transgenic tobacco seedlings for their stress tolerance

To assess the relative stress tolerance of various plants, WT and transgenic seeds ectopically expressing *OsMT1e*-*P* were germinated on MS medium supplemented with NaCl (100, 200 or 300 mM) or 5 mM CuSO_4_ or ZnSO_4_ (5 or 10 mM) or 5% PEG for imposing different abiotic stresses or onto plain MS medium that served as the experimental control. The seedlings were maintained under culture room conditions (28 ± 1°C, 16 h light/8 h dark) and their growth was monitored for 15 days under different stresses and fresh weight of surviving seedlings was measured.

### Leaf disc assay and measurement of chlorophyll contents

Leaf discs of 1 cm diameter were excised from healthy and fully expanded tobacco leaves of similar age from transgenic and WT plants were kept in half strength Hoagland media containing 200 mM NaCl or 5 mM CuSO_4_ or 10 mM ZnSO_4_ or 5% PEG. For cold stress, leaf discs were exposed to 4°C for 5 days while heat stress was given for 8 h at 42°C. Leaf discs kept in half strength Hoagland were taken as control. The chlorophyll content was measured spectrophotometrically after extraction in 80% acetone [63]. The experiment was repeated thrice with three different transgenic lines (n = 3).

### *In planta* histochemical estimation of H_2_O_2_

Accumulation of H_2_O_2_ was examined based on histochemical staining by 3, 3'-diaminobenzidine (DAB) as described earlier [48]. WT and transgenic leaves of 15 days old seedlings were kept in 200 mM NaCl stress for different time intervals (30 min, 1 h, 2 h, 4 h, 6 h and 21 h). These leaves were vacuum infiltrated into 1 mg/ml fresh DAB solution (pH 3.8) prepared in 10 mM phosphate buffer (pH 7.8) and placed in a plastic box under high humidity and light until brown spots were observed (5 to 6 h). The stained leaves were then fixed with a solution of 3:1:1 ethanol: lactic acid: glycerol and photographed. The experiment was repeated thrice with three different transgenic lines (n = 3).

### Testing transgenic tobacco plants for their tolerance towards salinity stress

In addition to the experiments at seedling stage, we carried out the assessment for the tolerance of transgenic plants when they are grown in the presence of salinity throughout their life cycle. For this purpose, the seedlings were transferred to earthern pots and grown in a greenhouse until maturity (16 h light/8 h dark and 25°C ± 2°C) either in absence or presence of 200 mM NaCl. Various parameters such as plant height, total fresh weight of plant, number of days to flower and pod weight were taken into consideration for assessment of salinity stress tolerance. Three plants from each transgenic line were randomly picked up for this analysis.

### EDXRF analysis

Three plants each of transgenic lines and WT grown under control or salinity stress conditions (as described above) were harvested and washed with distilled water three times. Plants were separated into root, lower leaf, upper leaf and pod. Equal amount of these plant tissue samples were dried at 105 °C in an oven, crushed to fine powder using a mortar-pestle grinder. Dry plant powder thus obtained was pressed by using 15-ton pressure and tablets of 100.0 mg were made. EDXRF measurements were performed on the EDXRF spectrometer (PANalytical, Netherland) with a Ge solid state detector. The source of X-ray was 100 keV Gadolinium tube which allows fluorescence efficiency for K-lines higher than for L-lines of elements. All samples were measured for a period of 2000 seconds on the sample holders made of Titanium rings. For relative quantitative analysis of element, Epsilon software was used. The experiment was repeated thrice with three different transgenic lines (n = 3).

### Statistical analysis

Data analysis was performed in Microsoft excel and analysis of variance (ANOVA) and presented as mean ± standard error (SE) of three biological replicates. Statistical analysis was performed using One-way-Analysis-of Variance (ANOVA). This was followed by Tukey’s post-hoc multiple comparison test using SPSS (version 19.0) for data statistics at different time points. Different letters were used (p < 0.05) to present statistically significant differences and similar letters were considered as statistically non significant.

## Abbreviations

MT: Metallothionein; ROS: Reactive oxygen species; Os: *Oryza sativa*; At: *Arabidopsis thaliana*; PCR: Polymerase chain reaction; DAB: Diaminobenzidine; WT: Wild type.

## Competing interests

The author(s) declare that they have no competing interests.

## Authors’ contributions

GK performed all physiological and histochemical analysis of transgenic plants. The bioinformatics related computing work was done by HRK. VP-S and SK involved in amplification, cloning and transformation. RJ and RK participated in all RNA extractions and transcriptome analyses, qRT-PCR experiments. SM helped in statistical analysis of the data. SLSP and AP made contributions to the conception of the study and AP in the preparation of the final draft of the manuscript. All authors read and approved the final manuscript.

## Supplementary Material

Additional file 1 Figure S1 Sequence and phylogenetic analysis of MT members in *O. sativa*. (**A**) Multiple alignment of the amino acid sequences of OsMT proteins of *O. sativa*. The OsMT1e-P sequence was from the *O. sativa* sp. Pokkali*,* while rest of the sequences was from *O. sativa* sp. japonica. The (*) above the sequences shows the conserved amino acid residues present in OsMT proteins. The figures were prepared using Jalview multiple alignment editor. (**B**) Rooted tree of OsMTs from *O. sativa* sp. japonica and OsMT1e-P from *O. sativa* sp. Pokkali. MUSCLE program was used for alignment, and PHYLIP was used for performing bootstrapping analysis and graphical presentation of the relationship among the OsMTs. (TIFF 3114 kb) Click here for file

Additional file 2 Figure S2 Small gene segment repeat on chromosome XII showing that certain regions may have got duplicated and diverged further during the course of evolution. The analysis was performed using Mummer software (Kurtz et al., 2004). (**A**) Mummer plot of 23.3 Mbp to 23.6 Mbp region of chromosome XII. (**B**) Dotplot analysis of 23.3 Mbp to 23.6 Mbp region of chromosome 12. Dotplot was plotted using *dottup* program using EMBOSS package. (TIFF 1444 kb) Click here for file

Additional file 3 Figure S3 Relative stress tolerance of WT and OsMT1e-P over expressing transgenic T_1_ generation tobacco plants at seedling level. (**A**) Leaf disc senescence assay for salinity stress tolerance in transgenic tobacco plants. Experiments were performed on three transgenic lines (L1, L2 and L7). Leaf discs of uniform size were floated on either NaCl solution (200 or 300 mM) for salinity stress or on water which served as control. Photographs were taken after 5 days of treatments. (**B**) K^+^/Na^+^ ratio of the leaves of the OsMT1e-P transgenic plants incubated in 200 mM and 300 mM NaCl. Data are means ± SE. Each data set represents an average of minimum three separate experiments. Bars with different letters are statistically significant and those with the same letters are not significantly different (p < 0.05). (TIFF 4089 kb) Click here for file

Additional file 4 Table S2 List of primers used for qRT-PCR analysis in the present study. (PDF 9 kb) Click here for file
