# Supplementary figures and images for: Clustered metallothionein genes are co-regulated in rice and ectopic expression of OsMT1e-P confers multiple abiotic stress tolerance in tobacco via ROS scavenging
Source: BMC Plant Biol. 2012 Jul 10;12:107. doi: 10.1186/1471-2229-12-107 (PMC3491035; doi:10.1186/1471-2229-12-107)

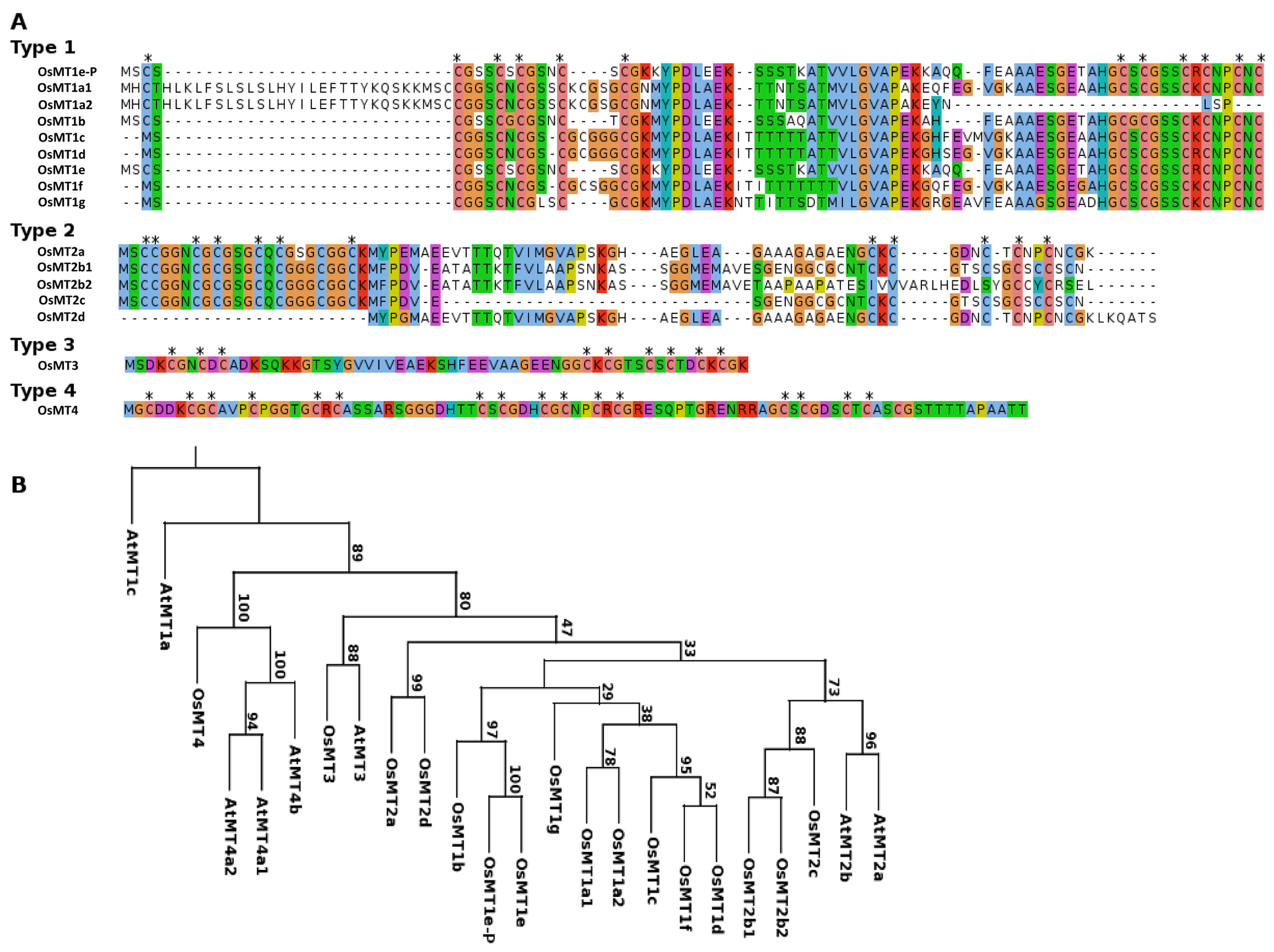

Supplement: Additional file 1 Figure S1 — Sequence and phylogenetic analysis of MT members in O. sativa. (A) Multiple alignment of the amino acid sequences of OsMT proteins of O. sativa. The OsMT1e-P sequence was from the O. sativa sp. Pokkali, while rest of the sequences was from O. sativa sp. japonica. The (*) above the sequences shows the conserved amino acid residues present in OsMT proteins. The figures were prepared using Jalview multiple alignment editor. (B) Rooted tree of OsMTs from O. sativa sp. japonica and OsMT1e-P from O. sativa sp. Pokkali. MUSCLE program was used for alignment, and PHYLIP was used for performing bootstrapping analysis and graphical presentation of the relationship among the OsMTs. (TIFF 3114 kb) [file 1471-2229-12-107-S1.tiff]

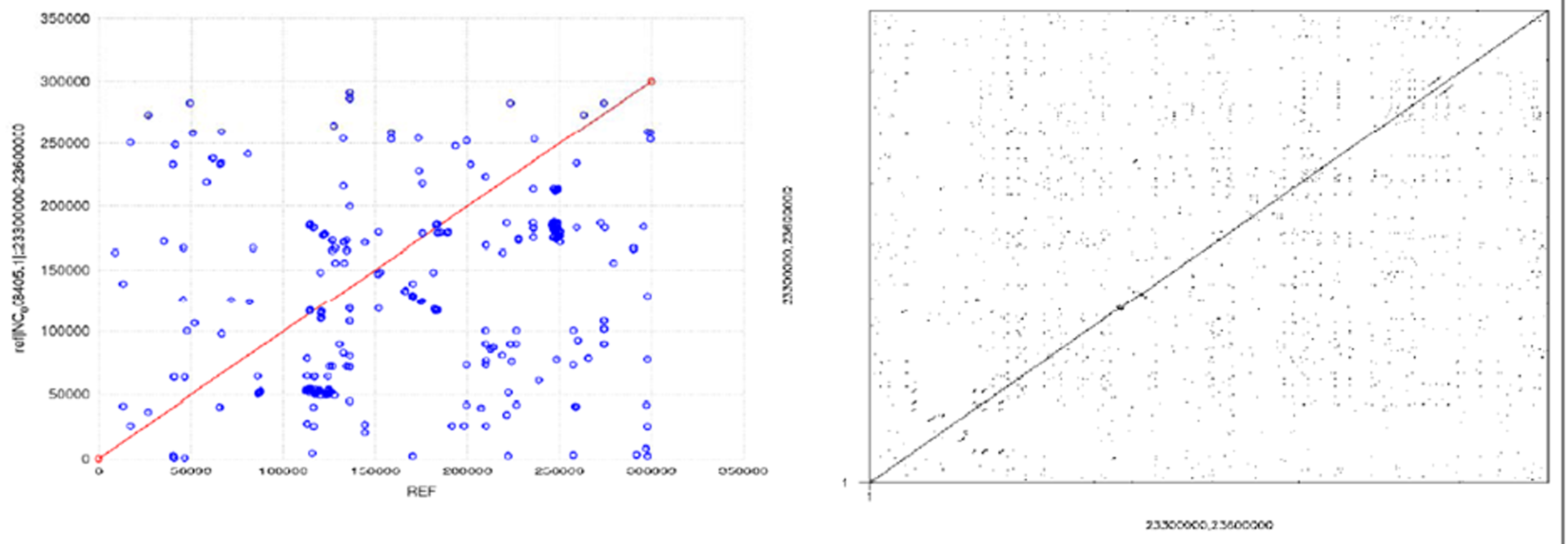

Supplement: Additional file 2 Figure S2 — Small gene segment repeat on chromosome XII showing that certain regions may have got duplicated and diverged further during the course of evolution. The analysis was performed using Mummer software (Kurtz et al., 2004). (A) Mummer plot of 23.3 Mbp to 23.6 Mbp region of chromosome XII. (B) Dotplot analysis of 23.3 Mbp to 23.6 Mbp region of chromosome 12. Dotplot was plotted using dottup program using EMBOSS package. (TIFF 1444 kb) [file 1471-2229-12-107-S2.tiff]

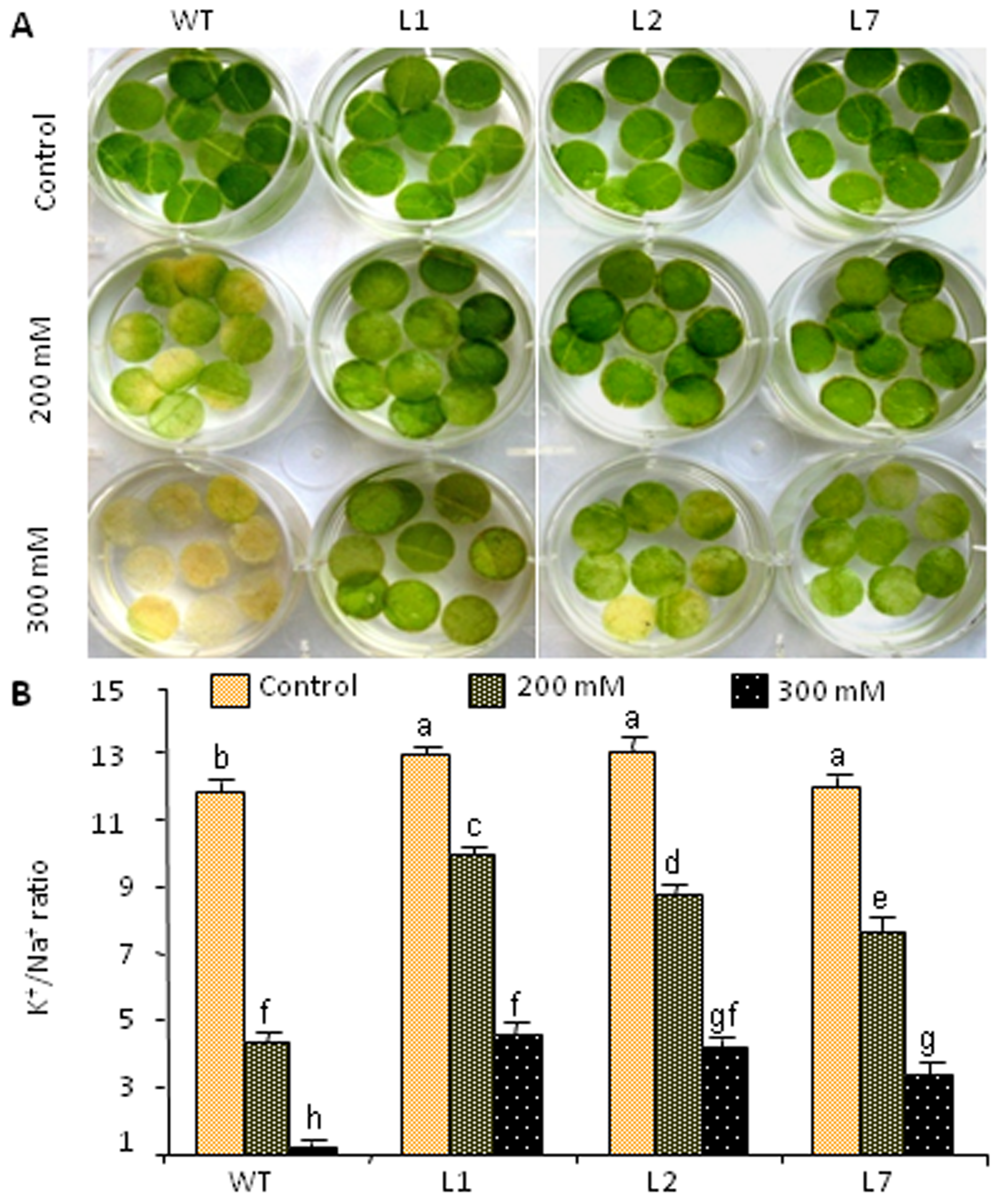

Supplement: Additional file 3 Figure S3 — Relative stress tolerance of WT and OsMT1e-P over expressing transgenic T1 generation tobacco plants at seedling level. (A) Leaf disc senescence assay for salinity stress tolerance in transgenic tobacco plants. Experiments were performed on three transgenic lines (L1, L2 and L7). Leaf discs of uniform size were floated on either NaCl solution (200 or 300 mM) for salinity stress or on water which served as control. Photographs were taken after 5 days of treatments. (B) K+/Na+ ratio of the leaves of the OsMT1e-P transgenic plants incubated in 200 mM and 300 mM NaCl. Data are means ± SE. Each data set represents an average of minimum three separate experiments. Bars with different letters are statistically significant and those with the same letters are not significantly different (p < 0.05). (TIFF 4089 kb) [file 1471-2229-12-107-S3.tiff]
