# Supplementary material for: Clustered metallothionein genes are co-regulated in rice and ectopic expression of OsMT1e-P confers multiple abiotic stress tolerance in tobacco via ROS scavenging
Source: BMC Plant Biol. 2012 Jul 10;12:107. doi: 10.1186/1471-2229-12-107 (PMC3491035; doi:10.1186/1471-2229-12-107)
Supplement: Additional file 4 Table S2 — List of primers used for qRT-PCR analysis in the present study. (PDF 9 kb) [file 1471-2229-12-107-S4.pdf]

Table 1: Members of metallothionein family in rice

Earlier proposed name	Gene	protein name	Tigr ID	Locus	Coordinate	Amino Acids
OsMT-I-4a	OsMT1a	OsMT1a1	12012.m07613	LOC_Os12g38270.1	23467876 – 23468536 (+)	108
		OsMT1a2	12012.m56497	LOC_Os12g38270.2	23467876 – 23468352 (+)	82
OsMT-I-1b	OsMT1b	OsMT1b	12003.m07212	LOC_Os03g17870.1	9936480 – 9937064 (+)	73
OsMT-I-4b	OsMT1c	OsMT1c	12012.m73910	LOC_Os12g38051.1	23350565 – 23349930 (-)	80
OsMT-I-4c	OsMT1d	OsMT1d	12012.m07616	LOC_Os12g38300.1	23481607 – 23482236 (+)	79
OsMT-I-1a	OsMT1e	OsMT1e	12011.m80109	LOC_Os11g47809.1	28269831 - 28270196 (+)	75
	OsMT1f	OsMT1f	12012.m07587	LOC_Os12g38010.1	23319560 – 23318879 (-)	79
	OsMT1g	OsMT1g	12012.m07615	LOC_Os12g38290.1	23479046 – 23479680 (+)	77
	OsMT2a	OsMT2a	12001.m07196	LOC_Os01g05650.1	2691774 – 2690331 (-)	83
OsMT-I-2c	OsMT2b	OsMT2b1	12005.m28027	LOC_Os05g02070.3	584010 – 584488 (+)	85
		OsMT2b2	12005.m27672	LOC_Os05g02070.4	584010 – 584488 (+)	58
OsMT-I-2b	OsMT2c	OsMT2c	12001.m13456	LOC_Os01g74300.1	43374103 – 43374519 (+)	81
	OsMT2d	OsMT2d	13101.m00538	LOC_Os01g05585.1	2665458-2664412 (+)	65
OsMT-I-3a	OsMT3a	OsMT3a	12001.m07661	LOC_Os01g10400.2	5475423 – 5476251 (+)	61
OsMT-II-1a	OsMT4	OsMT4	12010.m06732	LOC_Os10g39610.2	20838860 – 20839209 (+)	88
